# The Utility of Anatomical Liver Resection in Hepatocellular Carcinoma: Associated with Improved Outcomes or Lack of Supportive Evidence?

**DOI:** 10.3390/cancers11101441

**Published:** 2019-09-26

**Authors:** Michelle Ju, Adam C. Yopp

**Affiliations:** Department of Surgery, Division of Surgical Oncology, University of Texas Southwestern Medical Center, 5323 Harry Hines Boulevard, Dallas, TX 75390, USA; michelle.ju@utsouthwestern.edu

**Keywords:** hepatocellular carcinoma, hepatic resection

## Abstract

Hepatocellular carcinoma (HCC) is the second leading cause of cancer-related deaths worldwide. Surgical resection of HCC remains one of the mainstays of curative therapies and is associated with five-year overall survival rates approaching 60%. Despite improved perioperative outcomes, locoregional recurrence within the first two years following hepatic resection is of significant concern with recurrence rates of up to 50%. The use of anatomical resection surgical approaches, whereby the portal venous blood flow is ligated proximal to the tumor bed, is postulated to reduce recurrence rates due to reduction of micrometastatic disease. The aim of this review is to characterize the definition of an anatomical resection (AR) during partial hepatectomy, discuss the theoretical advantages of AR during hepatic resection for HCC, and to present evidence of the impact of AR on outcome measures in patients with HCC. Based on current data, there is a lack of conclusive evidence to support the universal use of AR in cirrhotic patients with HCC. A randomized clinical trial is warranted to further clarify the debate between AR versus non-anatomical resection (NAR) for HCC.

## 1. Introduction

Hepatocellular carcinoma (HCC) is the sixth most common cancer and the second leading cause of cancer-related deaths worldwide [1,2]. HCC most commonly occurs in a background of chronic liver disease with or without cirrhosis. Prognosis is dependent not only on tumor burden, but on underlying liver function and patient performance status as well. The heterogeneity seen in HCC patients with underlying liver dysfunction and a concomitant malignancy requires a multifaceted treatment approach. Surgical resection or partial hepatectomy, liver transplantation, and ablative therapies remain the only curative options in treating HCC with five-year survival rates in selected patients approaching 60% in most centers worldwide [3]. Due to heterogeneity of the patient population and low utilization of HCC screening, only 10–37% of patients are candidates for surgical resection at initial HCC diagnosis [4,5,6].

Advances in perioperative assessment of underlying liver function and radiologic extent of HCC tumor, surgical and anesthesia techniques, and postoperative care have led to significant decreases in morbidity and mortality following liver resection over the past two decades. Currently, the 30-day mortality following resection for HCC in high-volume centers is expected to be less than 5%, and there is a known association between volume and outcome following HCC diagnosis that is also seen in patients treated with surgical resection [7]. Despite improvements in perioperative outcomes, rates of recurrence following surgical resection for HCC remains a significant concern, with greater than 50% of patients exhibiting locoregional recurrence within five years [3]. Although most hepatic surgeons follow the dogma of tumor-free or negative resection margins as the controllable surgical factor to prevent tumor recurrence and preserve sufficient functional hepatic parenchyma to prevent perioperative hepatic failure, studies by Professor Masatoshi Makuuchi in 1985 advocated for the use of the anatomical resection surgical technique in HCC patients, whereby the tumor-free margin is independent of margin distance from the cut surface of the postresection hepatic parenchyma [8].

Although the concept of anatomical resection (AR) was first proposed nearly three decades ago, its use during the surgical resection of tumors in HCC patients has not been universally adopted. The aim of this review is to characterize the definition of an anatomical resection during partial hepatectomy, discuss the theoretical advantages of AR during hepatic resection for HCC, and to present evidence on the impact of AR on outcome measures in patients with HCC.

## 2. Defining Anatomical Resection and Description of Operative Techniques during Hepatectomy

Anatomical resection, first described by Makuuchi et al. in 1985 [8], is defined as the resection of the tumor together with the hepatic segment or subsegment, which includes tumor-bearing portal tributaries as well as a major branch of the portal vein and hepatic artery. Non-anatomic resection (NAR), also known as parenchymal-sparing resection, involves a less extensive liver resection than that of anatomic resection and is best defined as resection of a lesion regardless of the anatomical segment or section of the lobar anatomy. NAR includes limited resection or enucleation and offers the potential risk reduction of postoperative liver failure in patients undergoing resection for HCC, oftentimes in the background of a cirrhotic liver.

Anatomical resection involves either segmentectomy or sectionectomy—which includes bisectionectomy, hemihepetectomy, and trisectionectomy—and can be accomplished utilizing one of two surgical techniques: (1) Glissonean pedicle transection or (2) ultrasonically guided transection [9].

### 2.1. Glissonean Pedicle Transection Technique

Glissonean pedicle transection, first described by Couinaud for a left hepatectomy in 1985, was subsequently modified and expanded upon by Takasaki in 1998 [10] to describe a more limited hepatic resection including a subsegmentectomy based upon the division of the liver into four hepatic segments: right, middle, and left (the caudate lobe was not included in his original characterization). Anatomically, this division is based upon findings that demonstrate that the extrahepatic structures within the hepatoduodenal ligament—including the hepatic artery, portal vein, and bile duct—are sheathed in connective tissue or peritoneum and are indistinguishable from each other anatomically. The main “trunk” of the Glissonean pedicle expands at the hepatic hilum into two branches: the right and left primary branches. The right branch subdivides into two secondary branches (right and middle), and the left branch continues as a secondary branch extrahepatically. Utilizing the Brisbane terminology [11], the right secondary branch corresponds to segments 6 and 7, the middle secondary branch corresponds to segments 5 and 8, and the left secondary branch corresponds to segments 2, 3, and 4. The right hepatic vein is located between the right and middle secondary branches and the middle hepatic vein lies between the middle and left secondary branches. Each of the three segments are further subdivided into 6–8 smaller areas called “cone units” based on tertiary branches of the three main secondary branches.

According to the Glissonean pedicle classification as described by Takasaki, resection of HCC tumors within any of the three segments is done by first ligating the corresponding segmental branch of the Glissonean pedicle and then dissecting along the intersegmental plane of the appropriate hepatic vein, thus resecting the liver parenchyma while preserving the associated main hepatic vein.

Further subsegmental resection for tumors limited anatomically to discrete areas within the liver can be accomplished through the resection of one or more “cone units” within the segment by isolating tertiary branches and resecting selectively through a hilar or parenchymal approach.

The main limitation of the utilization of a Glissonean pedicle approach to achieve anatomical resection comes from in its use of subsegmental resections or resection of “cone units”, especially in segments 7 or 8 as defined above. Anatomical studies of cadavers demonstrate that there is wide variability in the intrahepatic origin of tertiary branches within the vast majority of Couinaud segments. This especially holds true in segments 7 and 8, where as many as four tertiary branches can supply one “cone unit”, thereby making selecting isolation difficult. Additionally, the isolation of subsegmental tertiary branches of the hepatic artery and portal vein, especially in the posterosuperior segments of the liver (segments 7 and 8), can lead to unnecessary parenchymal dissection in segments of the liver not in the planned transectional plane.

### 2.2. Ultrasonographic Guided Transection Technique

The use of intraoperative ultrasound (IOUS) scanning of the liver in conjunction with infusion of indigo carmine dye into the appropriate portal vein was first advocated by Makuuchi et al. in 1985 as a surgical technique to identify the landmarks allowing a targeted segmental or subsegmental area of resected liver [8]. In this technique, the hepatic artery is clamped at the hepatic hilum, blue dye (indigo carmine) is injected under IOUS guidance, and hepatic parenchymal transection is carried out from the liver surface inwards proceeding to the portal pedicle using the blue stained liver parenchyma as a guide. The dissection is carried out along the side of the associated main hepatic vein and the portal pedicle is ligated proximally, corresponding to the target area of the liver with the tumor [12].

The main limitations of IOUS-guided injection of dye as a technique for performing anatomical resection are that the procedure requires the surgeon to be facile with advanced IOUS techniques and unless arterial flow is occluded to the liver, reliable dye staining can be difficult to obtain.

The use of indocyanine green (ICG) fluorescent dye during hepatobiliary surgery has emerged over the past few decades and is now widely utilized [13]. IGC is injected intravenously, binds to plasma proteins, and remains in the vascular space until it is selectively taken up by the liver and then subsequently secreted into the bile. Although contrast enhanced IOUS remains the gold standard for liver mapping intraoperatively, portal hypertension resulting from cirrhosis might obstruct conventional liver mapping by ultrasonography. In these instances, fluorescence imaging can allow accurate visualization even in the presence of cirrhosis [14]. Additional benefits of ICG are that it is nontoxic and has been shown to allow accurate visualization of HCC tumors. However, tracking the stained plane during dissection of the liver parenchyma can also be difficult using ICG as the fluorescent dye within the target segment gradually disappears. Repeated injection of ICG or intermittent clamping the hepatic artery may be necessary for reducing washout of the dye and ensuring perpetual visualization of the segment [15].

### 2.3. What Is the Appropriate Surgical Technique for Anatomical Resection?

In surgical cases where tumor location requires either a right or left hemi- or extended hepatectomy or a sectional resection of the liver, the Glissonean pedicle approach is a relatively easy and safe method for accomplishing an anatomical resection due to the anatomical landmarks and extrahepatic location of the pedicles. However, if the tumor is positionally located where a segmental or subsegmental approach is warranted, the Glissonean pedicle approach is technically demanding, and a more useful approach is the IOUS-guided dye approach. For tumors located in segments 7 and 8, further away from the main portal pedicle, the IOUS-guided dye approach is generally more efficacious, especially in the hands of a surgeon experienced with IOUS.

The ability to utilize either approach depending on tumor location seen on appropriate preoperative contrast-enhanced imaging is key to performing an anatomical resection for HCC safely. Both techniques rely on liver parenchymal dissection along the right or middle hepatic veins—areas with high potential for catastrophic bleeding unless proper surgical and anesthetic technique with low central venous pressure is adhered to.

## 3. Theoretical Advantages of Anatomical Resection Surgical Technique for HCC

The high incidence of HCC recurrence following surgical resection may be explained by the high incidence of both intrahepatic metastasis and the multicentric occurrence of de novo HCC in the background of chronic liver disease [16,17]. Early recurrence, defined as recurrence within the first two years of resection, accounts for more than 70% of tumor recurrences and is presumed to be related to intrahepatic metastasis from the original resected tumor. Genetic and molecular studies of the clonal origin of primary and recurrent tumors have consistently demonstrated that early recurrence within the first two years of HCC tumor resection is likely to be associated with aggressive tumor pathological factors such as high tumor grade, microvascular invasion, and microsatellite lesions, whereas late recurrence is more likely related to underlying liver conditions such as the presence of cirrhosis and hepatitis activity [18,19].

The pathophysiology explaining early recurrence of HCC within the first two years of hepatic resection seems to be best explained by the hypothesis that HCC, unlike other primary or secondary liver tumor malignancies, has a high predilection for invasion into portal venous branches. This allows tumor cells to be disseminated via portal venous flow into other regions of the liver, forming tumor venous thrombi and subsequent satellite or new HCC lesions [17]. Theoretically, the utilization of anatomical resection would hinder dissemination of tumor cells throughout portal venous flow into the remnant liver and would be the surgical technique most apt to reduce early recurrence of HCC following resection. In 2018, Hidaka et al. published a retrospective analysis of 546 patients with HCC with microportal invasion (vp1), where they found that local recurrence occurred significantly more frequently in the NAR than the AR group [20]. However, this hypothesis has some limitations in explaining early recurrences as it assumes that tumor cells have not already left the tumor into the portal venous system as tumor circulating cells prior to resection.

## 4. Outcome Measures Following Anatomical Resection of HCC: The Evidence

Although utilizing an anatomical approach for HCC tumor resection should theoretically offer an advantage in outcomes compared to a non-anatomical approach aimed at preserving liver parenchyma to avoid postoperative liver failure in cirrhotic patients, the published data comparing the two approaches does not offer a consensus with regards to an advantage of one approach over the other.

To our knowledge, only one randomized controlled trial has been published examining the differences in outcomes between anatomical and non-anatomical approaches in HCC resection. In 2017, Feng et al. published a randomized trial corresponding to a single institution where 105 patients with HCC were randomized to either anatomical or non-anatomical hepatic resection with two-year local recurrence rate as the primary endpoint [21]. Local recurrence was defined as a recurrence occurring in the same hepatic section as the location of the initial primary tumor.

Although the use of anatomical resection was associated with a significantly decreased two-year local recurrence rate compared to non-anatomical resection (30% vs. 59%, *p* = 0.001, respectively), there was no difference between the approaches in either overall recurrence rate or overall survival. As concerns about postoperative complications may be one reason for the lack of universal adoption for anatomical resection, there were no differences in perioperative or postoperative complication rates between the two approaches. The main limitation of this study was that by utilizing the primary endpoint of local recurrence defined by the authors as within the same segment as the initial tumor, recurrences in different segments or extrahepatically would be censored in their results. This explains the lack of significance in recurrence-free survival between the two groups. As the hypothesis for the use of anatomical resection is postulated on the theory that anatomical resection reduces micrometastatic disease or tumor cells found in associated portal venous flow, recurrence limited to only the section of the liver that is removed is too limited as a primary endpoint. In addition, as most patients in this study had hepatitis B-related chronic liver disease and few cirrhotic patients, there are concerns over the applicability of the results to patients with chronic liver disease secondary to other etiologies.

Due to the paucity of randomized clinical trials comparing anatomical and non-anatomical resection for HCC, data on the relative benefit of AR versus NAR for HCC have largely been derived from retrospective clinical series. In turn, many of these reports have been limited due to sample size, which has impeded the ability to perform robust statistical analyses as well as reduced the generalizability of the findings. Thus, collation of individual case series into systemic review and meta-analyses methods have been utilized to discern differences between the two approaches. Since 2010, there have been two large systemic reviews with meta-analysis comparing anatomical resection versus non-anatomical resection for HCC utilizing nearly the same datasets. Surprisingly, although the two studies include data extracted from similar individual studies, the conclusions drawn vary based on included co-variates and primary outcomes.

**Figure 1:**
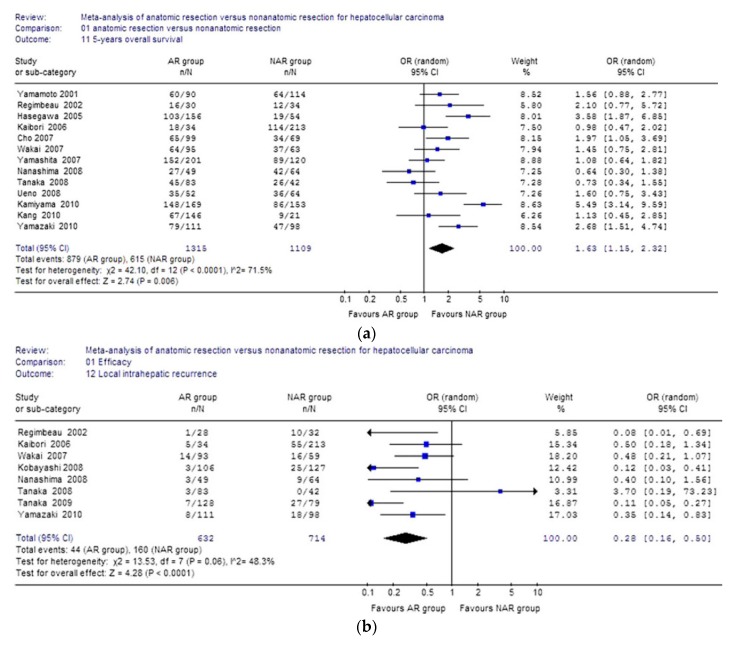
(**a**). Results of the Zhou et al. meta-analysis on 5-year overall survival. (**b**). Results of the Zhou et al. meta-analysis on local recurrence rate. Reprinted by permission from Springer Nature: Springer Langenbeck’s Archives of Surgery Meta-Analysis of Anatomic Resection Versus Non-Anatomic Resection for Hepatocellular Carcinoma, Zhou et al. (2011) [22].

In 2011, Zhou et al. published the first large meta-analysis comparing anatomical versus non-anatomical resection for HCC [22]. They analyzed the results of 16 retrospective studies conducted between 1996 and 2010 including 2917 total patients (1577 in anatomical and 1340 in non-anatomical groups) (Figure 1a,b). Primary outcomes were overall survival at 3 and 5 years, the rate of local intrahepatic recurrence, and disease-free survival at 3 and 5 years. Secondary outcomes were morbidity and mortality. The authors found that the AR group had significantly higher overall survival at 5 years (66.8% in AR group vs. 55.5% in NAR group, *p* = 0.006). In addition, the AR cohort had lower rates of local intrahepatic recurrence than the NAR cohort (6.9% vs. 22.4%, OR 0.28; 95% CI 0.16–0.50, respectively. Disease-free survival rates in the NAR group were also worse than in the AR group at both 3 years (34.5% vs. 52.1%) and 5 years (43.9% vs. 25.3%). There were no significant differences in overall mortality or perioperative complications between the two approaches.

**Figure 2:**
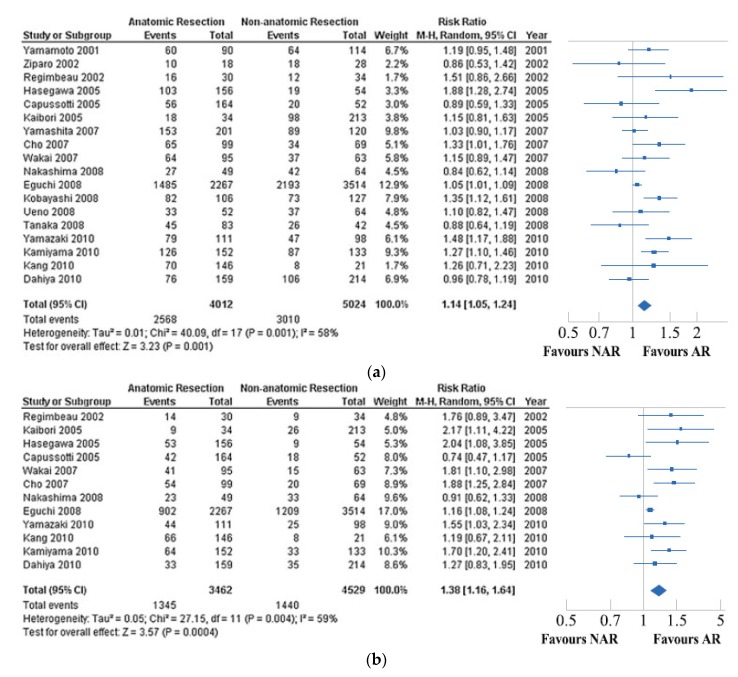
(**a**). Forest plot displaying the result of the Cucchetti et al. meta-analysis comparing 5-year overall survival. (**b**). Forest plot displaying the result of the Cucchetti et al. meta-analysis comparing 5-year disease-free survival of the anatomical resection (AR) group versus the non-anatomical resection (NAR) group. Reprinted by permission from Springer Nature: Springer Annals of Surgical Oncology A Comprehensive Meta-Regression Analysis on Outcome of Anatomic Resection Versus Non-Aanatomic Resection for Hepatocellular Carcinoma, Cucchetti et al. (2012) [23].

Utilizing many of the same extracted studies, Cucchetti et al. [23] also performed a meta-analysis comparing anatomical and non-anatomical resection for HCC in 2012. Not surprisingly, outcome measures including five-year overall survival and disease-free survival demonstrated similar results as the study by Zhou et al., with NAR having both worse overall survival disease-free survival compared to AR. Rates of postoperative complications were similar (Figure 2a,b). However, Cucchetti et al. performed a meta-regression of co-variates included in the 18 extracted studies. They demonstrated that differences in both overall and disease-free survival were mitigated not only by surgical approach but also by the underlying liver function. Using cirrhosis as a surrogate of underlying liver function, the authors demonstrated that the NAR cohort consisted of patients with a higher prevalence of cirrhosis, more advanced hepatic dysfunction, and higher prevalence of hepatitis C infection than the AR cohort. Each of these factors are individually associated with increased rates of recurrence following HCC resection in multiple published reports. This meta-regression demonstrated that inclusion of cirrhosis as a moderator of both five-year overall survival and disease-free survival reduced the residual heterogeneity to such an extent that technique of resection, anatomical or non-anatomical, was not significant.

**Figure 3:**
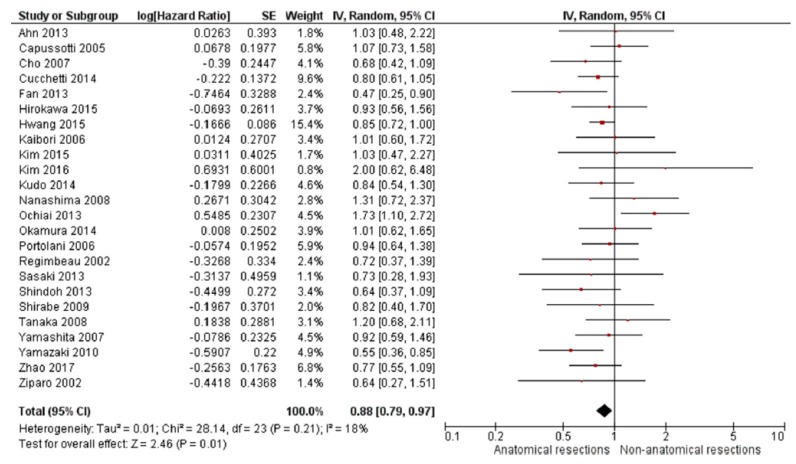
Five-year overall survival Moris et al. meta-analysis. Reprinted from EJSO, Vol 44/Issue 7, Dimitrios Moris et al., Anatomic Versus Non-Anatomic Resection for Hepatocellular Carcinoma: A Systematic Review and Meta-Analysis, pages 927–938, Copyright (2018), with permission from Elsevier [24].

**Figure 4:**
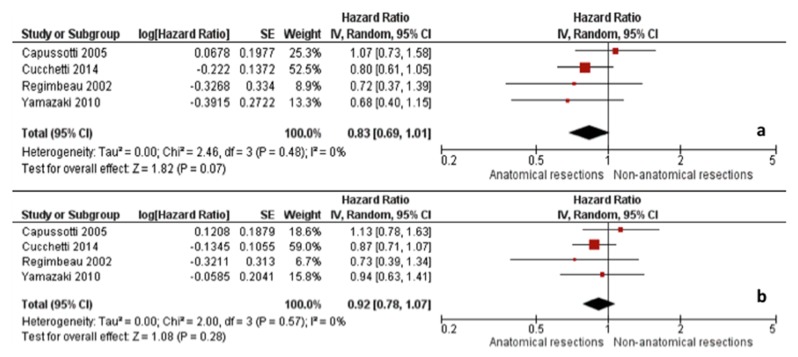
Moris et al. meta-analysis including only cirrhotic patients: (**a**) 5-year overall survival and (**b**) 5-year disease-free survival. Reprinted from EJSO, Vol 44/Issue 7, Dimitrios Moris et al., Anatomic Versus Non-Anatomic Resection for Hepatocellular Carcinoma: A Systematic Review and Meta-Analysis, pages 927–938, Copyright (2018), with permission from Elsevier [24].

A more recent meta-analysis from 2018 by Moris et al. also compared anatomical resection versus non-anatomical resection for HCC by extracting data from 43 studies and including 12,429 patients (6839 non-anatomical resections and 5590 anatomical resections) [24]. Consistent with the studies by Zhou et al. and Cucchetti et al., non-anatomical resection was associated with worse five-year overall and disease-free survival compared to anatomical resections when all patients were included (Figure 3). However, when only cirrhotic patients were analyzed, the benefit of anatomical resection on five-year survival (HR 0.83, 95% CI 0.69–1.01) and disease-free survival (HR 0.92; 95% CI 0.78–1.07) were similar to those of non-anatomical resection (Figure 4). Unfortunately, only four studies were available for inclusion in this subanalysis [7,25,26,27].

## 5. Conclusions

The theoretical advantage of an anatomical resection approach for HCC is in the potential reduction of micrometastatic disease within portal venous flow. Although a technically challenging operation with the potential of increasing rates of perioperative complications due to hepatic failure from the increased hepatic parenchyma volume resected, multiple studies have demonstrated no differences in morbidity or mortality compared to non-anatomical resection. However, the evidence for improved outcome measures outside of non-cirrhotic HCC patients is limited, and until a randomized controlled trial is conducted in cirrhotic only patients, the benefit in terms of overall survival and locoregional rates of recurrence anatomical resection for HCC remains unclear.
